# Candidiasis caused by *Candida kefyr *in a neonate: Case report

**DOI:** 10.1186/1471-2334-12-61

**Published:** 2012-03-18

**Authors:** Stefan Weichert, Konrad Reinshagen, Katrin Zahn, Gernot Geginat, Annebärbel Dietz, Anna Kristina Kilian, Horst Schroten, Tobias Tenenbaum

**Affiliations:** 1 Pediatric Infectious Diseases, University Children's Hospital Mannheim of Heidelberg University, Mannheim, Germany; 2 Department of Pediatric Surgery, University Hospital Mannheim of Heidelberg University, Mannheim, Germany; 3 Institute for Medical Microbiology and Hygiene, University Hospital Mannheim of Heidelberg University, Mannheim, Germany; 4 Department of Clinical Radiology and Nuclear Medicine, University Hospital Mannheim of Heidelberg University, Mannheim, Germany; 5 Pediatric Infectious Diseases, University Children's Hospital Mannheim of Heidelberg University, Theodor-Kutzer-Ufer 1-3, 68167 Mannheim, Germany

**Keywords:** Children, Candidiasis, Non-*albicans Candida *species, Urinary tract infection

## Abstract

**Background:**

Systemic *Candidia *infections are of major concern in neonates, especially in those with risk factors such as longer use of broad spectrum antibiotics. Recent studies showed that also term babies with underlying gastrointestinal or urinary tract abnormalities are much more prone to systemic *Candida *infection. We report a very rare case of candidiasis caused by *Candida kefyr *in a term neonate.

**Case Presentation:**

Renal agenesis on the left side was diagnosed antenatally and anal atresia postnatally. Moreover, a vesico-ureteral-reflux (VUR) grade V was detected by cystography. The first surgical procedure, creating a protective colostoma, was uneventful. Afterwards our patient developed urosepsis caused by *Enterococcus faecalis *and was treated with piperacillin. The child improved initially, but deteriorated again. A further urine analysis revealed *Candida kefyr *in a significant number. As antibiotic resistance data about this non-*albicans Candida *species are limited, we started liposomal amphotericin B (AMB), but later changed to fluconazole after receiving the antibiogram. Candiduria persisted and abdominal imaging showed a *Candida *pyelonephritis. Since high grade reflux was prevalent we instilled AMB into the child's bladder as a therapeutic approach. While undergoing surgery (creating a neo-rectum) a recto-vesical fistula could be shown and subsequently was resected. The child recovered completely under systemic fluconazole therapy over 3 months.

**Conclusions:**

Candidiasis is still of major concern in neonates with accompanying risk factors. As clinicians are confronted with an increasing number of non-*albicans Candida *species, knowledge about these pathogens and their sensitivities is of major importance.

## Background

Systemic *Candida *infections in children are of major concern in preterm infants, neonates with risk factors and in immunocompromised children [1,2]. Further risk factors such as use of central venous catheters, longer use of broad spectrum antibiotics and use of parenteral nutrition contribute as well [2]. Over the last decade non-*albicans Candida *species are emerging as causative pathogens for systemic *Candida *infections in children [3,4]. Here, we report of a candidiasis caused by *Candida kefyr *in a term neonate.

## Case Presentation

**Figure 1:**
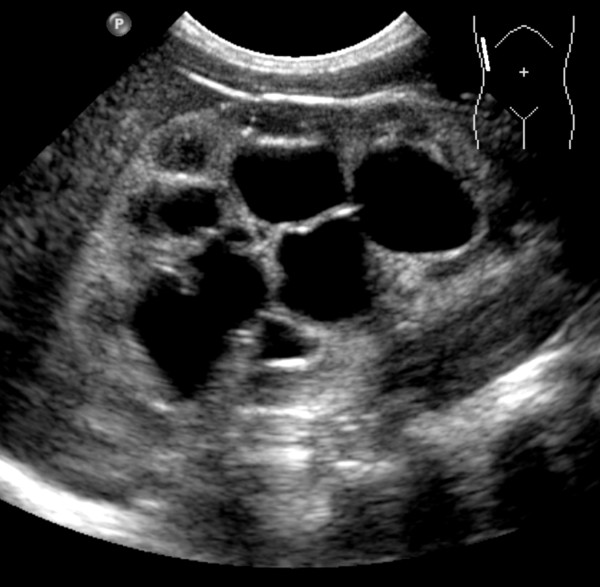
**Renal ultrasound**. Hyperechogenic renal parenchyma due to persistent *Candida *pyelonephritis and massive pelviectasis.

After an uneventful birth anal atresia was observed and a vesico-ureteral-reflux (VUR) grade V was detected by cystography. Renal agenesis on the left side was already diagnosed antenatally. The first surgical procedure, creating a protective colostoma, on day 2 was uneventful. The child was treated with intravenous cefotaxime for 10 days and was put hereafter on cefixime prophylaxis. On day 21 the patient developed an urosepsis caused by *Enterococcus faecalis *which was treated with piperacillin according to the antibiogramm. After initial improvement the child deteriorated again 10 days after initiation of antibiotic treatment. Antibiotic therapy was changed to imipenem, gentamicin and vancomycin. A lumbar puncture showed normal results, but the urine analysis revealed significant fungal growth (10^6 ^CFU/mL). Primary isolation was performed on *Candida *Chrom™ Agar (BD) which yielded growth of large rough pink colonies, resembling *Candida krusei*. Therefore systemic antifungal therapy was initiated with liposomal amphotericin B (AMB). Further identification of the suspected non-*albicans Candida *species was performed by biochemical identification using the API 20 C AUX (BioMerieux) biochemical identification panel which yielded excellent identification (probability > 99.9%, profile number 7220300031). This biochemical result was confirmed by sequencing of the internal transcribed spacer (ITS) regions using primer pairs ITS 1 and ITS-4 (ITS1: 5-TCCGTAGGTGAACCTGCGG-3, ITS4: 5-TCCTCCGCTTATTGATATGC-3 and V9D:5-TTAAGTCCCTGCCCTTTGTA-3 and LS266:5-GCATTCCCAAACAACTCGACTC-3, respectively) [5]. Both primers span the complete ITS1, 5.8S, and ITS2 regions. A databank search of the amplified sequences revealed 99% and 100% homology with the ITS region from *Kluyveromyces marxianus *which represents the teleomorph form of *Candida kefyr*. Susceptibility testing against fluconazole, amphotericin B (AMB), and caspofungin was performed by ellipsometer test ("E-test") and showed a minimal inhibitory concentrations (MIC) of 0,25 μg, 0,047 μg, and 0,25 μg, respectively. All tests were repeated two times with similar results. The inoculum for susceptibility testing was generally performed by pooling of 10-20 individual colonies. No macrocolonies were observed in the inhibition zone of the E-test. Further subplating and antibiotic susceptibility testing of individual colonies in order to detect antibiotic susceptibility variants was not performed. Species-specific susceptibility breakpoints for *Candida kefyr *have neither been published by the clinical laboratory institute (CLSI) nor by the European committee on antimicrobial susceptibility testing (EUCAST). Therefore we used the EUCAST breakpoints for *Candida albicans *for the interpretation of the MICs obtained with the *Candida kefyr *isolate. According to these breakpoints the isolate was susceptible to all three antifungal agents tested. After the availability of susceptibility data antifungal treatment was changed to fluconazole. Although the child improved clinically, a significant candiduria persisted and renal ultrasound showed persistent signs of *Candida *pyelonephritis (Figure 1). Blood culture results turned out to be negative. An initial contrast enema did not show a connecting fistula between bladder and rectum. But due to the clinical course a fistula was suspected and surgical repair of the anorectal atresia was performed. While undergoing surgery (creating a neo-rectum) a recto-vesical fistula was found and subsequently was resected. Since high grade reflux was prevalent in our patient AMB (1 μg/mL) was successfully instilled into the child's bladder twice daily over 7 days. *Candida kefyr *was isolated for the last time after 7 days of treatment, afterwards, all tested urine cultures remained sterile and a second blood culture remained sterile as well. The child recovered completely under systemic fluconazole therapy (8 mg/kg/day) over 3 months.

## Discussion

The high burden of systemic *Candida *infection in children with risk factors led to a significant increase in fluconazole use over the last decades, which was accompanied by an increased incidence of non-*albicans Candida *species. Interestingly, susceptibility of the main causative pathogen *Candida albicans *to fluconazole remains stable [3,4]. In contrast, a recent study showed only 82% susceptibility of all isolated non-*albicans Candida *species to fluconazole [3]. Data regarding susceptibilities of antifungal agents against *Candida kefyr *are limited. The isolated *Candida kefyr *from our patient was fully sensitive to fluconazole. In a 10.5-year world-wide surveillance study resistance to fluconazole ranged from 3.3% in the first 4 study years to 1.7% for all *Candida kefyr *isolates in the last 3 study-years [4]. So far, good susceptibilities of AMB against most non-*albicans Candida *species were shown, although country specific differences were observed [4,6,7]. According to a study from Pfaller et al. the susceptibility of *Candida kefyr *to amphotericin B appears to be quite low (4 of 10 isolates were susceptible at ≤ 1 μg/ml) [8]. A study conducted in Germany involving mainly adult patients showed an increased MIC of AMB for 9% of all *Candida kefyr *isolates [9], whereas a more recent study from Spain showed no increased MIC of AMB [7].

Although our patient had recurrent infections due to *Candida kefyr *and had clinical symptoms of systemic disease the pathogen *Candida kefyr *was only isolated from urine cultures and not from blood cultures or other sites. Our patient suffered from grade V reflux, that may led to an ascending kidney infection. However, it is reported that amongst clinical signs for systemic disease isolated candiduria may be the only indication for candidaemia. Studies confirmed that blood cultures are 40-75% false negative in patients with candidiasis, as demonstrated in patients with autopsy proven candidiasis [10,11]. In addition to clinical signs of systemic disease, our patient had renal involvement as well, such as parenchymal changes on ultrasound. An ascending infection would be expected to result in isolated pelvicalyceal disease, and it is known that haematogenous spread is the most common route for renal candidiasis [12]. Therefore, it is conceivable, that patients may have transient candidaemia that may lead to organ involvement. Nevertheless, it is known that blood cultures are often no longer positive when renal candidiasis becomes manifest [13]. As candiduria is regarded as a risk factor for invasive candidiasis [14] clinicians should be aware of this, even though blood cultures might remain negative.

Up to now *Candida kefyr *is considered as not pathogenic to healthy individuals, but has been discussed as an emerging pathogen in patients with risk factors. Pediatric data are sparse, reporting isolation of *Candida kefyr *from 1.8% to 4% of all isolated *Candida *species from mainly preterm und low birth weight neonates [15,16]. In adults *Candida kefyr *has been reported to cause systemic *Candida *infection in patients with neutropenic leukemia [17] and in a woman with underlying heart disease [18]. Very recently *Candida kefyr *was described as a pathogen causing invasive fungal enteritis in a patient with underlying haematological disease following bone marrow transplantation [19]. Of note, Sendid et al. report a twofold detection rate of *Candida kefyr *isolates from adult patients in oncohematology wards compared to patients in other wards (4.8% vs. 1.9%) [20]. Up to now, it is not known why *Candida kefyr *is found more often in these patients. Induced selection of *Candida kefyr *following antimicrobial therapy or prophylaxis is discussed, as well as factors that might influence gastrointestinal homeostasis in favour of *Candida kefyr *[20]. Furthermore, as *Candida kefyr *is commonly found in dairy products, dietary habits might influence or promote colonization and subsequent infection in patients as well [21].

## Conclusion

As clinicians are confronted with an increasing number of non-*albicans Candida *species, knowledge about these pathogens and their sensitivities is of major importance. In children with recurrent candiduria systemic infection and organ involvement should be ruled out, even though blood cultures might remain negative.

## Consent

Written informed consent was obtained from the patient's guardian for publication of this case report and any accompanying images. A copy of the written consent is available for review by the Editor-in-Chief of this journal.

## Competing interests

The authors declare that they have no competing interests.

## Authors' contributions

SW took care of the patient and drafted and wrote the manuscript. KR took care of the patient and contributed to the draft of the manuscript. KZ took care of the patient and helped to the draft of the manuscript. GG performed and interpreted all mentioned microbiological methods and revised the manuscript. AD performed and confirmed identification of the mentioned pathogen. AK performed the ultrasound imaging studies and contributed to the draft of the manuscript. HS contributed in coordinating the manuscript and to the draft of the manuscript. TT took care of the patient and coordinated and edited the manuscript. All authors have read the manuscript and approved its final version.

## Pre-publication history

The pre-publication history for this paper can be accessed here:

http://www.biomedcentral.com/1471-2334/12/61/prepub
